# “It was pain. That’s it. It was pain.” Lack of oral health care among otherwise healthy young adults living with HIV in South Africa: A qualitative study

**DOI:** 10.1371/journal.pone.0188353

**Published:** 2017-12-22

**Authors:** R. Frederick Lambert, Catherine Orrell, Jessica E. Haberer

**Affiliations:** 1 Harvard School of Dental Medicine, Harvard University, Boston, MA, United States of America; 2 Desmond Tutu HIV Foundation, Institute of Infectious Disease and Molecular Medicine, University of Cape Town, Cape Town, South Africa; 3 Center for Global Health, Massachusetts General Hospital, Boston, MA, United States of America; 4 Harvard Medical School, Harvard University, Boston, MA, United States of America; University of Queensland, AUSTRALIA

## Abstract

**Introduction:**

The purpose of this study is to understand engagement with and availability of dental services among people living with HIV in a low-income community of South Africa.

**Methods:**

In depth qualitative interviewing was used to collect data, which was analyzed using an inductive content analytical approach. The study was conducted in Gugulethu, a township community located outside of Cape Town, South Africa. Local public sector health services provided free of charge are the main source of primary health and dental care for this population. Participants included South African adults (age 18–35) recently diagnosed with HIV who had a CD4 count >350 cells/mm^3^.

**Results:**

Many participants had little to no experience with dental care, did not know which health care providers are appropriate to address oral health concerns, were not aware of available dental services, utilized home remedies to treat oral health problems, harbored many misperceptions of dental care, avoided dental services due to fear, and experienced poverty as a barrier to dental services.

**Conclusions:**

Our findings suggest that integration of oral healthcare into medical care may increase patient knowledge about oral health and access to care. Leveraging the relatively robust HIV infrastructure to address oral disease may also be an effective approach to reaching these participants and those living in resource poor communities generally.

## Introduction

Oral diseases are both common and easily preventable or treatable early in disease progression [1]. Lack of access to quality oral health services may lead to impaired eating, self-esteem, social interactions, and speech, among other conditions [2]. The adverse effects of poor oral health compounded with systemic disease in developing countries is debilitating to individuals, causing pain, disfigurement and even death, as well as to communities, negatively impacting local economies [2,3]. Recently, the World Health Organization (WHO) recognized the need to address the growing epidemic of poor oral health, acknowledging poverty, inequality, and systemic disease as indicators [4].

The South African health care system suffers from issues with access to care generally, especially among populations living in resource-poor communities. Access to oral health services is no exception. Provision of dental services is unequal and there is a shortage of dentists working in the public setting; while more than 80% of the South African population (about 56 million) relies on public dental services, less than a quarter of dentists in the country work in the public sector.[5] In South Africa, poor access to care and low socio-economic status, among a variety of other determinants, contributes to oral disease such as dental caries. According to the National Children’s Oral Health Survey, the Western Cape region has the population with the greatest need for dental care in South Africa (80% of children needed restorative care for treatment of caries) perhaps due to higher sugar intake in urban areas [6,7,8]. Lack of emphasis on preventive dental care and education, including among children, may lead to South African patients presenting late in dental disease progression where the only treatment option is extraction [8]. Furthermore, reports suggest a high level of untreated caries among all age groups, a high prevalence of missing teeth among the adult population and extraction the most common dental treatment available for South Africans [9,10]. South African populations that suffer from high prevalence of infectious diseases such as HIV/AIDS, often also suffer high burden of dental disease, which may be exacerbated by inequality in access to care [11,12].

People living with HIV/AIDS (PLHA) are at higher risk of oral diseases such as fungal infections, periodontal disease, and leukoplakia, among others [13,14]. Often, oral manifestations of HIV/AIDS are the earliest sign of immunocompromise [9,15]. While numbers of PLHA on antiretroviral therapy (ART) are improving, those on ART may also be at risk of adverse oral health effects from the medication including decreased salivary flow rate leading to higher risk of dental caries [16,17,18]. Appearance of oral lesions such as candidiasis may also signal treatment failure in PLHA on ART [9]. In accordance with WHO guidelines for universal access to ART regardless of CD4 count, increasing numbers of PLHA are entering care at earlier stages of disease progression [19,20]. Provision of oral health care should therefore focus more attention on preventive measures in order to avoid decline of oral health status among these high-risk individuals [10,21].

Here, we present a qualitative study of experiences accessing oral healthcare in a resource poor community of South Africa among otherwise healthy young adults living with HIV and recently initiating ART. We aimed to explore their experiences with oral health services early in HIV infection. The data presented here may provide valuable knowledge of the strengths and shortcomings of dental care services, as well as reveal potential interventions to increase access to and delivery of quality, preventive oral health services to a population at higher risk of oral disease.

## Methods

### Overview

‘Examining HIV Treatment Adherence During Early Disease’ (NCT 02419066; the “parent study”) is an observational cohort study involving objective measurement of adherence behavior and correlates of adherence in early- and advanced-stage disease, among pregnant and non-pregnant individuals, in Uganda and South Africa. As a sub-study in South Africa, we conducted qualitative interviews of young adults to learn about motivations and barriers to accessing HIV testing and treatment, as well as other services including oral health care. Findings related to HIV testing and treatment have been previously reported [22]. This manuscript focuses on the participants’ experience with oral health care.

### Ethical approval

This study was approved by Partners Healthcare Institutional Review Board, Boston, MA (Protocol#: 2015P000841), Harvard University Faculty of Medicine Office of Human Research Administration, Boston, MA (Protocol#: IRB15-1948), University of Cape Town Faculty of Health Sciences Human Research Ethics Committee, Cape Town, South Africa (HREC REF: 078/2015), and the Western Cape Provincial Health Research Committee (Reference: WC_2015R26_119). Written consent was obtained from all participants. All procedures performed in studies involving human participants were in accordance with the ethical standards of the institutional and/or national research committee and with the 1964 Helsinki declaration and its later amendments or comparable ethical standards.

### Study site

Qualitative interviews took place between June and November 2015 in Gugulethu (population approximately 100,000, primarily low socioeconomic status), a township community located outside of Cape Town, South Africa. Local public sector health services provided free of charge are the main source of primary health and dental care for this population and those residing in the surrounding townships such as Crossroads, Nyanga, and Khayelitsha. Residents of Gugulethu may also seek medical services in neighboring townships as well. In Gugulethu, free dental services are only provided at Nyanga Clinic. The study recruited from the Hannan Crusaid Treatment Centre (HCTC) and Vuyani Clinic, which are primary health care facilities broadly representative of the spectrum of primary care services across similar townships surrounding Cape Town. Neither HCTC nor Vuyani Clinic provides dental care services.

### Sampling and recruitment

The study population consisted of participants between 18 and 35 years of age, drawn from an on-going, longitudinal ART adherence study, ‘Examining HIV Treatment Adherence During Early Disease’ (NCT 02419066), which required that participants were initiating ART for the first time at enrollment. Participants with CD4 count (350 cells/mm3) who had recently started or made the decision to start ART were included. PLHA were excluded from this qualitative study if they were infected perinatally, pregnant, or unable to provide informed consent. Participants were selected by convenience sampling of participants meeting eligibility criteria. Pregnancy and perinatal infection likely raise unique concerns best studied separately. Participants were recruited as they came into the clinic and those that fit inclusion criteria were asked to participate–all recruited participants agreed to the interview. Informed consent was obtained from all individual participants included in the study.

### Qualitative data collection

A single, hour long, in-depth, semi-structured, qualitative interview was conducted with each participant at the time of enrollment into the parent study. Open-ended questions elicited motivations and barriers to dental care. A R100.00 (approximately $7.00) incentive was issued to each participant. Interviews were conducted in a private location in the local language, Xhosa by a bilingual (English/Xhosa) research assistant from the community. Interviews were audio-recorded with participant permission and directly transcribed into English (without first transcribing in Xhosa) [23].

### Data analysis

Factors influencing access to oral healthcare were identified using a conventional content analytical approach [24]. Content pertaining to oral health care within the transcripts was reviewed. Using NVivo 11, two investigators (RFL, JEH) thematically coded the first five interviews and a codebook was created. This codebook was iteratively updated to represent new, recurrent themes, pulled directly from the text data, which were then applied to all 25 transcripts. The influence, relevance and meaning of each theme were discussed among the authors and were then grouped into descriptive categories. Recruitment and interviewing continued until thematic saturation was achieved. The participant’s thoughts and beliefs that comprise each theme were highlighted by specifically chosen quotations from the interviews.

## Results

### Participant characteristics

Twenty-five participants were invited to participate this study and all agreed to the interview. Participant characteristics are shown in Table 1. The median participant age was 28 years old; 6 men and 19 women were interviewed. Per enrollment criteria, all participants were living with HIV and had recently decided to begin ART. All had CD4 counts >350 cells/mm^3^ and none reported symptoms related to HIV infection.

**Table 1:** Participant characteristics stratified by gender.

**Demographics**	**Men**	**Women**	**Total**
**Total**	6	19	25
**Median Age**	25 (IQR = 22–28)	30 (IQR = 23–32)	28 (IQR = 23–31)
**Children cared for by participant**			
0	5	2	7
≥1	1	17	18
Employment*			
Employed	3	10	13
Unemployed	2	7	9
Student	1	3	4
**Demographics**	**Men**	**Women**	**Total**
**Total**	6	19	25
**Median Age**	25 (IQR = 22–28)	30 (IQR = 23–32)	28 (IQR = 23–31)
**Children cared for by participant**			
0	5	2	7
≥1	1	17	18
Employment*			
Employed	3	10	13
Unemployed	2	7	9
Student	1	3	4

*One participant was both a student and employed.

### Overview of qualitative findings

Analysis revealed several themes describing participants’ experience with dental health care: (1) No experience with dental care, (2) Use of medical clinics due to lack of knowledge about oral health services, (3) Reliance on home remedies to treat oral health problems, (4) Misperceptions of dental care, (5) Avoidance of dental services due to fear, and (6) Poverty as a barrier to dental services. Each theme is presented in detail below.

### No experience with dental care

Many participants had never been to a dentist nor had any oral health problems addressed. Some were aware that dental service existed, but did not know how or where to access them. These participants had not yet experienced symptomatic dental issues that necessitated urgent treatment. They were unaware or not concerned with any asymptomatic oral disease. They also had not sought preventive dental care.

“I never went to the dentist, and I don’t know anything about the dentist, although I know the dentist [exists]” (Female, 27)

Participants’ responses suggested that dental care played little, if any, role and has thus far had no relevance to their lives.

### Use of medical clinics due to lack of knowledge about oral health services

When asked where participants would choose/have chosen to go to address an oral health concern, many participants were unsure or said they would go to a medical clinic.

“Interviewer: If you can experience a tooth pain what will you do?

Participant: I will still go to KTC, Gugulethu day hospital.” (Male, 28)

“…I asked someone, where to take the tooth out, so someone told me that I must go to the clinic next to Gugulethu Mall, in 3A, and I’m not sure if that doctor is a dentist or what I’m not sure.”

(Female, 26)

Additionally, some participants were not aware of which conditions would be appropriate for dentists to treat. For example, one participant stated that he would go to a medical doctor, not a dentist, to address gingival bleeding.

“Interviewer: Will you ever go to the dentist?

Participant: No, I will go to the doctor not the dentist, and anyway I had a problem of bleeding gums not tooth pain.” (Female, 30)

Lack of knowledge surrounding both accessing care and understanding which symptoms demand treatment by a dental professional reflected the lack of experience with oral health services among this population. Importantly none of the medical centers where they went offered dental services.

### Reliance on home remedies to treat oral health problems

Rather than seek care from any professional provider, several participants described home remedies or over the counter analgesics used to alleviate oral health problems such as toothaches.

“First thing I do when I have a tooth problem, I use boiled water with salt in it and gargle and spit, or put Colgate on the painful tooth. And if the pain persists I would go to a doctor to maybe get it extracted.” (Female, 21)

These remedies served as both initial therapies, which facilitated a delay in seeking professional care and sometimes encouraged participants to avoid seeking professional treatment all together.

“I have never gone to see a doctor yet when I have a toothache. I wait it out and just take some pain blockers and stay home.” (Female, 25)

For some participants, home remedies were the preferred treatment for dental concerns. For others, home remedies were used due to lack of knowledge of other available options for treatment.

### Misperceptions of dental care

Almost every participant believed that the primary indication for seeking dental care was tooth pain and the only treatment option extraction. One participant, when asked what she knows about dentists, replied explicitly that they extract teeth.

“Interviewer: What do you know about the dentist?

Participant: the only thing I know about the dentist is that they are taking the teeth out.” (Female, 22)

Another participant said that she expects the dentist to remove the tooth that is causing pain and that the procedure should be painless.

“Interviewer: What can you expect from the dentist?

Participant: I will expect the tooth to be taken out, but it mustn’t be sore/painful when they take it out.” (Female, 26)

The anticipated treatment and the treatment received by participants who had knowledge or experience with oral health services was almost unanimously extraction. There was a notable absence of commentary on dental services accessed for treatment of other indicators of oral disease such as bleeding gums.

### Avoidance of dental services due to fear

The expectation of extraction was a source of fear for many participants. Some stated that the fear of extraction caused them to avoid oral healthcare.

“Interviewer: What discouraged you not to seek dental care?

Participant: Because I’m afraid of taking the tooth out.” (Female, 24)

Another participant was afraid of the possible complications associated with extractions.

“You can criticize me; I am just plain scared of a tooth extraction. People say tooth extraction is something else and lead to other things.” (Female, 25)

The fear of dental treatment was sometimes overcome due to the pain that dental conditions may cause.

“Interviewer: What encouraged you to go to the dentist?

Participant: It was pain. That’s it. It was pain.” (Female, 31)

For some participants, past experiences involving extractions caused fear of future dental care. For others, although they had never seen a dentist in the past, fear of potential extraction prevented them from seeking care.

### Poverty as a barrier to dental services

The decisions to both seek dental care and choose between public and private dental providers often involved the price of treatment. Inability to afford private dental care (perceived as higher quality) and/or the failure of the health care system to offer a range of treatment and fund it accordingly was a barrier to care for some participants. In some cases, cost was an important barrier to seeking dental care. One participant suggested that he/she would choose to go to the medical clinic because the care is free.

“I can just go to the [medical] clinic… because everything is free there (participant laughing)” (Female, 23)

One participant voluntarily explained, when asked about his experience with medical care in general, that he was finally able to regularly see a private dentist because he received free medical aid (the equivalent of health insurance), presumably covering both medical and dental care.

“Interviewer: And why did you start seeing a dentist?

Participant: Because I had free medical aid dude. Yhoo!” (Male, 23)

Prior to receiving aid, he had never seen a dentist. In this case, the participant explained that regularly seeing a dentist was important to him, but cost had previously prohibited care.

“No, because I knew, that I have to go to the dentist regularly you know, but I didn’t have the money. Yhoo those people, they charge you. So now I have medical aid, so now I can go to the guy.” (Male, 23)

Conversely, for this participant, medical care, even with the medical aid covering costs, was only accessed as a reaction to illness.

“[I go] To the doctor, when I’m sick, man. I don’t get sick that much, like twice or three times a year. I go regular to the dentist though. I have a dentist I go to a lot.” (Male, 23)

Another participant suggested that he questioned the quality of care provided by public providers and because of that, he was willing to pay the expense of seeing a private dentist.

“I am going to go to a private dentist that you pay out of pocket because I think it would be better…They seem to have more care than the public dentist. For example the hole left by my tooth [which was extracted by a public dentist] is still painful even now.” (Male, 22)

Although a public dental clinic does exist locally in Gugulethu, cost was still reported as a barrier to seeking dental care.

## Discussion

**Fig 1:**
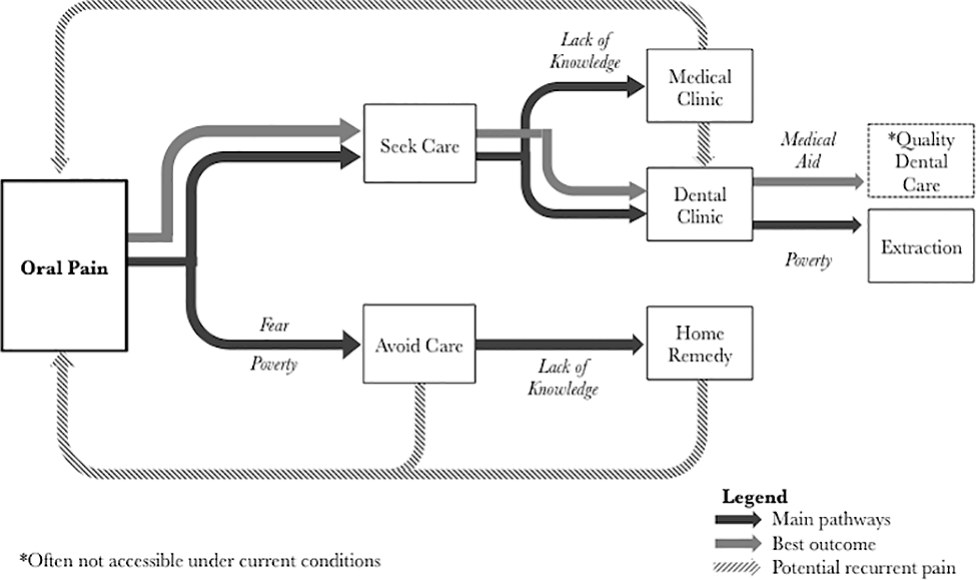
Descriptive model of participant experiences addressing oral pain and progressing from oral health symptoms to care.

Qualitative interviews of young adults living with HIV in South Africa indicate that a lack of access to high quality dental care services (e.g., beyond extractions and including preventative care) results in unnecessary pain and use of ineffective treatments and/or inappropriate services. The progression of symptoms and care experienced by participants is depicted by the descriptive model: Fig 1. Preventive dental care from public sources appears to be largely unavailable to this high-risk population. The data herein shows that participants only have access to minimal dental services–if they receive dental care at all. This scenario leads to individuals waiting until pain causes them to seek emergent care (i.e. extraction). If motivated to address the dental pain, some participants used home remedies, or sought care from public medical clinics. Only the participants who sought care at public health dental clinics received care, and treatment always consisted of extraction. Primary barriers to dental services included poverty (both participant’s lack of ability to pay for quality dental care and/or lack of resources to provide quality dental care, beyond extractions, by the national health care system) and fear of extraction due to past experience or conventional wisdom. Sometimes this anticipation encouraged participants to utilize home remedies thus delaying care or even avoid dental care all together. Interestingly, many barriers to care commonly cited in the literature describing similar populations such as transportation, crime, and medical mistrust were not mentioned by participants in this context. These structural barriers to care may have been less relevant to the participants of this study because they displayed an ability to access care for HIV.

The descriptive model (Fig 1) presented offers several areas of potential intervention. Lack of awareness of oral health care and reliance on home remedies for dental pain may be addressed through increased educational efforts. Focused programs providing education and oral health promotion pertaining to preventive dental care both at home and in the dental and/or medical clinic may serve to improve oral health and perhaps dispel myths of dental treatment that prevent access to care. An adult education program in the United States employed behavior modification principles to improve the oral health of participants living with HIV and observed an improvement in periodontal disease and an increase in self-reported preventive oral health habits such as brushing and flossing [25]. In China, an educational program implemented in kindergarten classes demonstrated a reduction in decay-missing-filled index and a significant increase in daily brushing [26]. Long-term follow-up suggests that school based education programs do have lasting effects on oral hygiene [27]. Educational programs should be tailored to the socioeconomic and cultural norms of each population.

The data presented here suggests that education should be coupled with changes in care delivery in order to address the fear and misperceptions exhibited by many participants due to the nature of care rendered by public dental clinics in the region [8]. A focus on preventive dental care delivery may help to reduce the numbers of extractions performed and therefore change the perception of public health dental services perhaps leading to an increase in care utilization. For those that require curative dental care, adoption of atraumatic restorative treatment, an inexpensive alternative treatment for dental caries, has demonstrated a reduction in anxiety and may be useful in addressing the fear of extraction that exists as a barrier to dental care among this group [28].

Participants were also unaware of where to find public dental services that could be provided to them, with most choosing to seek care at medical clinics where dental services are not provided. Those who access dental care at medical clinics may not ever receive the care they need if they do not subsequently seek care with a dentist. Misunderstandings of best uses of health services may benefit from improved communication campaigns by public health services. In addition to inadequate healthcare, physical separation of dental and medical care may lead to loss of productivity from additional work days missed in order to seek care at the dental clinic after first going to a medical clinic. Patient support in the form of patient navigators may increase patient awareness of services (medical and dental) currently available to them and encourage them to seek preventive care [29,30].

Many strategies may be utilized in order to begin to dismantle the barrier between medical and dental care services [2]. Physicians, nurses, and other providers, such as community health workers or dental hygienists, should be trained to both perform simple screens for oral diseases and to refer for appropriate care [13]. For example, if a physician upon oropharyngeal exam notes severely decayed teeth, he or she should refer to the appropriate public dental clinic for care. Similarly, dentists can use very simple diagnostics, such as taking blood pressure, to screen for chronic diseases such as hypertension, HIV, and/or diabetes and refer for management [10,11,31,32]. Colocation of medical and dental professionals would allow patients who arrive with an oral health complaint to be treated on site [33].

Utilization of infrastructure already in place, such as HIV clinics, to also counsel and treat oral disease may lead to improved access and outcomes [34]. Many have suggested that the relatively robust HIV response can be adapted to address non-communicable diseases (NCDs), such as hypertension and diabetes [35,36]. Oral disease should be added to this list. The test and treat strategy for HIV was developed to reach those who may not be aware of their HIV status and bring them into treatment [37]. Those suffering from NCDs may share similar characteristics in that they are not aware of the conditions affecting them and could benefit from risk factor identification and counseling similar to what is already in place in the HIV testing and treatment model [38]. The data herein suggests that policies encouraging integration of oral health promotion along with other NCDs into the HIV response infrastructure could benefit PLHA, in addition to others in their community, by streamlining and expediting care from diagnosis to treatment.

In addition to integrative measures, financial concerns must also be addressed [39]. Some participants only needed financial coverage (i.e. dental care included in medical aid/insurance) to motivate them to access preventive and regular dental care. Inclusion of dental care into basic medical aid coverage may encourage patients to access dental care more regularly and help to prevent dental disease. In Soweto, for example, attendance at public dental clinics increased after implementation of free dental coverage [40]. Our data supports this finding and suggests that expansion of such interventions may find success in increasing access to necessary preventive dental care among this population. Adoption of a preventive care model may actually be a more cost effective strategy and may facilitate expansion of coverage [4]. In 2015, the South African government proposed a restructuring of the healthcare system, including the Nation Health Insurance (NHI) program, focusing on prevention and health promotion which would begin to address some of the issues faced by many of the participants [41].

While access to oral health care is of particular importance to this cohort due to their HIV status, lack of HIV symptoms to date suggests that their experiences with oral health delivery may be generalizable to the community overall. The barriers and motivators reported by the participants are likely not unique to PLHA and any interventions implemented to address them would likely be beneficial to the community as a whole.

While many studies examine the oral manifestations of HIV/AIDS and inequalities in oral health, we are the first (to our knowledge) to explore access to quality oral healthcare among this particular demographic. The study design is limited by enrolling only those patients who have initiated ART. Additional studies are needed from a community-based sample to gain a deeper understanding of the barriers to dental care including those who are not living with HIV, PLHA who have not yet been diagnosed, or PLHA who have been diagnosed but are not on ART. Because participants did not explicitly describe how medical providers addressed their oral health needs, further investigation of care provided by medical clinics for dental complaints or referrals provided to patients at medical clinics is warranted. While a convenience sample of 25 individuals is relatively small and may not be representative of the larger population, it was sufficient to achieve saturation of identified themes.

## Conclusion

In depth qualitative data from otherwise healthy young adults living in a resource poor community in South Africa highlight the need for integrated, streamlined preventive oral healthcare. Our results suggest that there is a need for programs that overcome fear and misperceptions of oral health services. Additionally, participant experiences illuminate the detrimental consequences that the separation of medical and dental services has on patient care. Expansion of HIV infrastructure to tackle NCDs including oral disease may be a viable solution, benefitting both people living with and without HIV.
